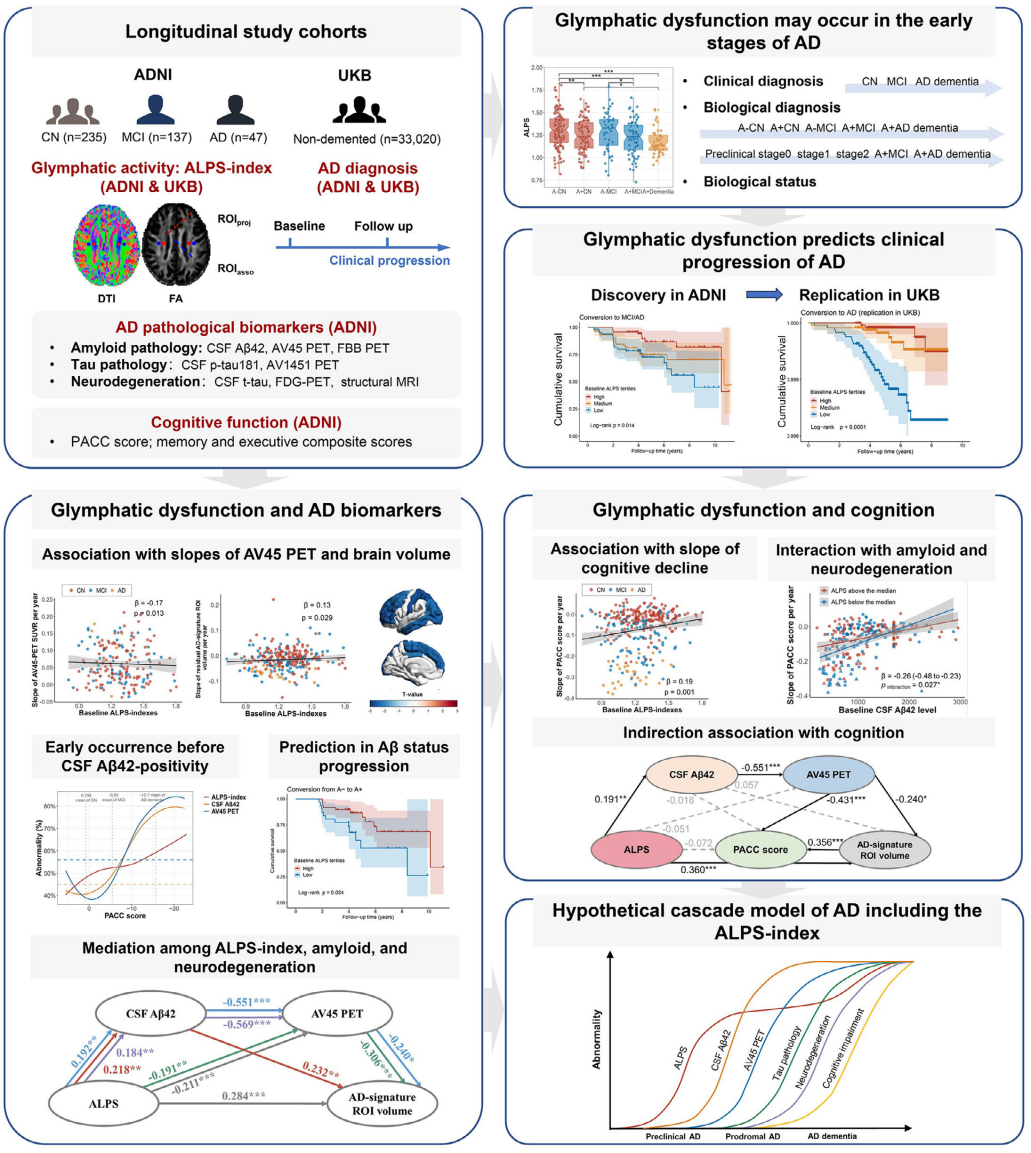# Glymphatic system dysfunction predicts amyloid deposition, neurodegeneration, and clinical progression in Alzheimer’s disease

**DOI:** 10.1002/alz.084295

**Published:** 2025-01-03

**Authors:** Shu‐Yi Huang

**Affiliations:** ^1^ Huashan Hospital, Fudan University, Shanghai China

## Abstract

**Background:**

Although glymphatic function is involved in Alzheimer’s disease (AD), its potential for tracking the pathological and clinical progression of AD and its sequential association with core AD biomarkers is poorly understood.

**Method:**

Whole‐brain glymphatic activity was measured by diffusion tensor image analysis along the perivascular space (DTI‐ALPS) in participants with AD (n = 47), mild cognitive impairment (n = 137), and normal controls (n = 235) from the Alzheimer’s Disease Neuroimaging Initiative.

**Result:**

Decreased ALPS‐index was observed in AD dementia, prodromal AD, and preclinical AD patients. Lower ALPS‐index was significantly associated with faster changes in amyloid PET burden (AV45 PET) and AD‐signature ROI volume, higher risk of amyloid‐positive transition and clinical progression, and faster rates of amyloid‐ and neurodegeneration‐related cognitive decline. Furthermore, the associations of ALPS‐index with cognitive decline were fully mediated by amyloid PET and brain atrophy.

**Conclusion:**

Glymphatic failure may precede amyloid pathology, and predicts amyloid deposition, neurodegeneration, and clinical progression in AD.